# The Relationship Between Social Capital and Sleep Duration Among Older Adults in Ghana: A Cross-Sectional Study

**DOI:** 10.3389/ijph.2023.1605876

**Published:** 2023-06-29

**Authors:** Jonathan Aseye Nutakor, Lulin Zhou, Ebenezer Larnyo, Alexander Kwame Gavu, Irfan Mir Chohan, Stephen Addai-Dansoh, Debashree Tripura

**Affiliations:** ^1^ School of Management, Jiangsu University, Zhenjiang, China; ^2^ Center for Black Studies Research, University of California, Santa Barbara, CA, United States; ^3^ Department of Educational Administration, College of Education, University of Saskatchewan, Saskatoon, SK, Canada

**Keywords:** older adults, social capital, sleep quality, sleep duration, sub-Saharan Africa

## Abstract

**Objective:** This study aims to investigate the connection between social capital and sleep duration among older adults in Ghana, as limited research has been conducted to explore this relationship.

**Methods:** This study utilized Wave 2 data from a sample of Ghanaian older adults from the World Health Organization Study on Global AGEing and Adult Health (SAGE). Self-reported data on social capital and sleep duration were compiled. Using ordered logistic regression, the relationship between social capital and sleep duration was examined.

**Results:** Older adults who did not participate in social activities showed the strongest association with the risk of short sleep (*p* < 0.05). Our study found that older adults who sleep for shorter periods tend to report better sleep quality. There was no correlation between medium and long sleep durations and social capital.

**Conclusion:** This study underscores the importance of more research to truly understand the complex connections between older adults’ social participation, sleep, and health. It also has important implications for the promotion of good sleep in aging populations.

## Introduction

Aging is related to changes in sleep patterns and an increased risk for sleep disorders [1]; therefore, sleep health among older adults is a growing concern. Research indicates that older adults frequently suffer from insomnia, sleep apnea, restless leg syndrome, and circadian rhythm abnormalities [2]. These sleep disturbances can have severe health effects, including an increased risk of falls, cognitive impairment, and a compromised immune system [3, 4]. In addition, chronic medical disorders and the usage of specific medications might affect sleep in the elderly [5]. It is advised that this group adhere to a regular sleep schedule, maintain a pleasant sleep environment, participate in physical exercise, and avoid stimulants such as caffeine and electronic screens before bedtime [6].

Xiao et al. [7] conducted a study in China to investigate the relationship between social capital and sleep health. Their findings suggest that individuals with higher social capital tend to have better sleep health, while those with lower social capital are at a higher risk of developing sleep disorders. The study highlights the importance of social connections and support in promoting healthy sleep habits, particularly in the context of a rapidly changing social landscape in China. Social capital refers to the resources and networks that individuals have access to as a result of their interactions with others, including friends, family, and community members. Studies have indicated a correlation between greater levels of social capital and improved sleep quality and fewer sleep disruptions [8, 9]. This may be because social ties provide support and a sense of belonging, which may reduce stress and anxiety and improve feelings of safety and security. On the other hand, low levels of social capital have been associated with poor sleep quality, an increase in sleep disruptions, and an elevated risk for sleep disorders [10, 11]. In addition, factors such as loneliness, social isolation, and lack of social support might have a poor effect on sleep health [12]. For instance, persons who experience feelings of isolation may have trouble falling or staying asleep owing to stress and anxiety. Those with strong social ties, on the other hand, may sleep better owing to heightened sensations of safety and security. According to a study by Kawachi et al. [13] in the United States, social capital is associated with self-reported health when socioeconomic status is taken into account. Overall, the association between social capital and sleep health outcomes underlines the significance of social ties and support in supporting healthy sleep [14].

Sleep disorders can lead to physical and mental exhaustion, disturbed moods, and impaired concentration, which can have a negative impact on family life, professional activities, and social interactions [2]. Studies have shown that not getting enough sleep increases the risk of stroke, heart disease, high blood pressure, diabetes, obesity, falls, death [15–17] and cancer [18, 19]. Recent statistics show that 7.7 million older adults in the US are socially isolated [12].

Despite evidence linking a lack of social support to negative sleep outcomes [20], the vast majority of prospective studies have focused on social support in the workplace, and these have either failed to control for or completely ignored participants’ sleep duration [20]. No prior study has assessed this connection in an older Sub-Saharan African population, and few studies on social capital and sleep duration among the elderly have been conducted in Anglo-Saxon and Asian populations [21–26]. As a result, the purpose of this study is to investigate the association between social capital and sleep duration among older adults in Ghana. This study employed data from the World Health Organization’s Study on Global AGEing and Adult Health—wave 2 to investigate the association that exists between social capital and sleep duration among older adults in Ghana. To the best of our knowledge, this is the first study in Ghana and sub-Saharan Africa to investigate the relationship between social capital and sleep duration in older adults.

## Methods

For this study, cross-sectional data were taken from the second wave of the World Health Organization Study on Global AGEing and Adult Health (SAGE). The SAGE study aimed to provide a comprehensive picture of the health and wellbeing of older adults globally and to inform efforts to promote healthy ageing and address the health needs of older populations. SAGE was carried out between 2014 and 2015 in six nations that fall within the low-and middle-income categories (China, Ghana, India, Mexico, Russia, and South Africa) [27]. We concentrated on the data that was gathered in Ghana since it was most relevant to the goals of this study. This data comprises individuals aged 50 and older. Ghana employed a stratified multi-stage cluster design with a nationally representative sample of individuals aged 50 and above. The administrative regions of Ashanti, Brong Ahafo, Central, Northern, Western, Greater Accra, Volta, Northern, Upper East, and Upper West as well as the type of residence (urban/rural) were stratified in the study sample [28]. This resulted in the formation of twenty different strata. Consequently, 251 enumeration areas (EA) were used as the primary sampling units (PSU) [28]. Participants in the interview were adults over the age of 50 from twenty households, as well as adults between the ages of 18 and 49 from four households. For those who were unable to participate in an interview due to health or cognitive issues, a proxy questionnaire was sent and filled out on their behalf. All interviewers who took part in the study obtained the necessary official training. Because the questionnaire was originally written in English, it was translated into several local languages to accommodate respondents who were unable to understand English [29, 30]. This means that the study includes participants from different ethnic and linguistic backgrounds, which is a strength of the study. Additionally, translating the questionnaire into local languages ensures that the questions are easily understood by all participants, regardless of their level of education or proficiency in English.

### Measures

To assess social capital, three different variables were constructed. These include social support, social participation, and trust [31]. The determinants of social capital were derived from a combination of seventeen separate questions. To determine whether or not the respondents had a trusting relationship with the individuals (friends, neighbors, and family) in their immediate environment, questions about trust were posed to them. Participants were asked whether they think that people can be trusted, whether they have someone they can trust and confide in, and whether they trust their neighbors, coworkers, and strangers (Supplementary File). Three of the five trust-measuring questions included original responses such as 1) to a very great extent, 2) to a great extent, 3) neither great nor small, 4) to a small extent, and 5) to a very small extent. To create a binary “yes” or “no” variable, responses 1, 2, and 3 were considered as “yes” (indicating trust to some extent) and responses 4 and 5 as “no” (indicating lack of trust). With the remaining two questions, original responses were coded as 1) yes/can be trusted, and 2) no/cannot be too careful. Response 1 was considered as “yes” (indicating that most people can be trusted/having someone to trust and confide in) and response 2 as “no” (indicating that one cannot be too careful in dealing with people/lacking someone to trust and confide in). In addition, questions on social participation focused on the participants’ involvement with family, friends, neighbors, and public gatherings. Participants were asked how frequently they attended public meetings, clubs, religious services, went out, worked with other people in their neighborhood, visited friends in their homes, and engaged with community leaders, friends, and coworkers in the previous 12 months. Original responses included 1) never, 2) once or twice a year, 3) once or twice a month, 4) once or twice a week, and 5) daily. To recode these responses into a binary “yes” or “no” response, responses 1 and 2 were considered as “no” (indicating infrequent attendance) and responses 3, 4, and 5 as “yes” (indicating regular attendance). Concerning the respondents’ access to social support, the question of whether or not they had persons with whom they could discuss their emotional needs, issues, or challenges was posed. Participants were asked how frequently they lack companionship, feel excluded, and sense isolation from others. Original responses were divided into four categories: 1) never, 2) rarely, 3) sometimes, and 4) often. To recode these responses into a binary “yes” or “no” variable, responses 1 and 2 were considered as “no” (indicating sufficient companionship) and responses 3 and 4 as “yes” (indicating a lack of companionship).

The amount of time spent sleeping by each participant the night before the research was the primary outcome of this investigation. During the course of the interview, the participants were asked the following question: “How many hours did you sleep last night?” For analysis, we classified the replies into three categories: fewer than 6 h, 6–8 h, and more than 8 h [28, 32]. Throughout the course of the research, we will refer to the group that sleeps for less than 6 h as short sleepers, the group that sleeps for 6–8 h as medium sleepers, and the group that sleeps for more than 8 h as long sleepers.

The analysis of this study took into account several covariates, including gender, age, residence, marital status, education level, household income quintile, chronic diseases (including stroke, diabetes, hypertension, and depression), lifestyle variables (including tobacco use, alcohol consumption, and body mass index), the quality of sleep, activities of daily living (ADL), and cognitive functioning. These were selected because prior research has established a connection between them and the amount of time spent sleeping [28, 33] as well as social capital [34, 35]. For a total of 21 assets, income quintiles were determined based on household ownership of durable items, home characteristics, and access to services (improved water, sanitation, and cooking fuel) [36]. To create the quintiles, a two-step random effect probit model was utilized. First, an asset ladder was created based on the endorsement rates of the various assets. This ladder was then used to place households on a similar scale, depending on asset ownership. As a result, a continuous income score is generated, from which quintiles are formed [36]. Self-reporting and validated symptom reporting served as the basis for the chronic condition study data. The interviewers inquired as to whether or not the respondent had been diagnosed with any of the following chronic conditions: stroke, diabetes, hypertension, or depression [29]. Data on alcohol and tobacco use was based on self-reported responses to questions on whether respondents had ever consumed alcohol or smoked tobacco. Body mass index (BMI) was calculated for each participant using their weight (kg) and height (m), and it was then divided into three categories: underweight, normal weight, overweight, and obese. Self-reported sleep quality was measured and classified as a binary outcome, with good/very good against other (moderate/poor/very poor). The physical functioning of the respondents was measured based on their self-reported activities of daily living. ADL refers to the fundamental personal activities involved in daily living. These basic self-care routines, which include walking, eating, dressing, using the restroom, and taking a shower, are taught to us as children [27]. The overall scores ranged from 0 to 10, with higher ADL scores indicating poorer physical functioning in older adults. In this study, cognitive function was evaluated objectively using a variety of cognitive tests (forward and backward digit spans; immediate and delayed verbal recall; and verbal fluency). To represent the respondents’ overall cognitive function, a total cognitive score was created. Greater values indicated greater functional cognition [29].

### Statistical Methods

In this study, the sample was characterized by the use of descriptive statistics. We used the following modelling strategy to construct ordered logistic regression models to investigate the connection that exists between social capital and the amount of time spent sleeping. In total, there were four different models. Trust, social participation, and social support were components of Model 1. In addition to trust, social participation, and social support, we adjusted for gender, age, residence, marital status, education level, and household income in model 2. In model 3, we made adjustments for tobacco use, alcohol consumption, and body mass index. Model 4 included adjustments for stroke, diabetes, hypertension, depression, sleep quality, ADL, and cognitive functioning. This analysis was carried out separately for each of the three different time frames of sleep. For all of the analyses, Stata/SE software (StataCorp College Station, TX) was utilized. If the value of p is less than 0.05, then the findings of the study are regarded as significant.

## Results

Table 1 contains the statistics that provide a summary of the data. The participants in this study are all 50 years old or older, making the total number of participants in this study 1,063. The total amount of time spent sleeping, on average, was 8.8 h. The majority of the respondents were male, making up 56.8% of the total. In terms of age, around 39.1% of respondents were under the age of 60, 38.1% were between the ages of 60 and 69, and 22.7% were beyond the age of 70. In comparison to their contemporaries who reside in urban areas, older individuals who live in rural areas made up more than half (55.1%) of those who responded to the survey. It was found that older adults who were currently married made up the majority (61.5%), followed by those who were widowed (19.6%), separated or divorced (15.1%), never married (2.8%), and cohabiting (0.9%). It is interesting to note that the majority of the respondents (25.8%) had not completed any schooling beyond primary school, as opposed to the minority (6.7%) who had completed college, university, or postgraduate study. The majority of respondents, approximately 36.5% of the total, belonged to the lowest income quintile, while 18.6% belonged to the highest income quintile. In terms of chronic diseases, those individuals who reported having experienced or been diagnosed with any of the following conditions were in the minority: depression (0.8%), diabetes (4.4%), hypertension (15.9%), and stroke (1.8%). In addition to this, the respondent’s lifestyles were evaluated based on their consumption of tobacco and alcohol. Alcohol and cigarettes were never used by more than half of the respondents (59.9% and 91.6%, respectively). Nine hundred and nineteen of the respondents, or 86.4%, assessed the quality of their sleep as either good or very good.

**TABLE 1:** Summary statistics of the studied variables (World Health Organization Study on Global AGEing and Adult Health, Ghana, 2014–2015).

Variable	*N* = 1,063 *n* (%)	Short sleep (22) *n* (%)	Medium sleep (144) *n* (%)	Long sleep (897) *n* (%)
Trust score, mean (SD)	2.13 (0.74)	2.15 (0.99)	2.15 (0.80)	2.13 (0.73)
Social Participation score, mean (SD)	2.71 (0.87)	2.30 (0.96)	2.73 (0.99)	2.72 (0.85)
Social Support score, mean (SD)	1.26 (0.52)	1.08 (0.23)	1.22 (0.52)	1.28 (0.53)
Gender				
Male	604 (56.82)	11 (1.82)	79 (13.08)	514 (85.10)
Female	459 (43.18%)	11 (2.40)	65 (14.16)	383 (83.44)
Age				
50–59	416 (39.13)	11 (2.64)	65 (15.62)	340 (81.73)
60–69	405 (38.10)	7 (1.73)	54 (13.33)	344 (84.94)
70–79	188 (17.69)	4 (2.13)	21 (11.17)	163 (86.70)
80+	54 (5.08)	0 (0.00)	4 (7.41)	50 (92.59)
Residence				
Urban	477 (44.87)	13 (2.73)	80 (16.77)	384 (80.50)
Rural	586 (55.13)	9 (1.54)	64 (10.92)	513 (87.54)
Marital status				
Never married	30 (2.82)	0 (0.00)	6 (20.00)	24 (80.00)
Currently married	654 (61.52)	10 (1.53)	93 (14.22)	551 (84.25)
Cohabiting	10 (0.94)	1 (10.00)	1 (10.00)	8 (80.00)
Separated/Divorced	160 (15.05)	5 (3.12)	19 (11.88)	136 (85.00)
Widowed	209 (19.66)	6 (2.87)	25 (11.96)	178 (85.17)
Education				
Less than primary school	275 (25.87)	5 (1.82)	46 (16.73)	224 (81.45)
Primary school	206 (19.38)	6 (2.91)	25 (12.14)	175 (84.95)
Secondary school	255 (23.99)	5 (1.96)	23 (9.02)	227 (89.02)
High school	233 (23.99)	5 (1.96)	40 (15.69)	210 (82.35)
College/University/Postgraduate degree	72 (6.77)	1 (1.39)	10 (13.89)	61 (84.72)
Household income				
Lowest	389 (36.59)	11 (2.83)	53 (13.62)	325 (83.55)
2	86 (8.09)	2 (2.33)	8 (9.30)	76 (88.37)
3	162 (15.24)	3 (1.85)	22 (13.58)	137 (84.57)
4	228 (21.45)	3 (1.32)	32 (14.04)	193 (84.65)
Highest	198 (18.63)	3 (1.52)	29 (14.65)	166 (83.84)
Stroke				
Yes	20 (1.88)	0 (0.00)	3 (15.00)	17 (85.00)
No	1,043 (98.12)	22 (2.11)	141 (13.52)	880 (84.37)
Diabetes				
Yes	47 (4.42)	1 (2.13)	10 (21.28)	36 (76.60)
No	1,016 (95.58)	21 (2.07)	134 (13.19)	861 (84.74)
Hypertension				
Yes	170 (15.99)	5 (2.94)	37 (21.76)	128 (75.29)
No	893 (84.01)	17 (1.90)	107 (11.98)	769 (86.11)
Depression				
Yes	9 (0.85)	0 (0.00)	3 (33.33)	6 (66.67)
No	1,054 (99.15)	22 (2.09)	141 (13.38)	891 (84.54)
Tobacco				
Yes	89 (8.37)	2 (2.25)	16 (17.98)	71 (79.78)
No	974 (91.63)	20 (2.05)	128 (13.14)	826 (84.80)
Alcohol				
Yes	426 (40.08)	12 (2.82)	59 (13.85)	355 (83.33)
No	637 (59.92)	10 (1.57)	85 (13.34)	542 (85.09)
BMI				
Underweight	128 (12.04)	3 (2.34)	12 (9.38)	113 (88.28)
Normal weight	566 (53.25)	11 (1.94)	63 (11.13)	492 (86.93)
Overweight	223 (20.98)	2 (0.90)	39 (17.49)	182 (81.61)
Obese	146 (13.73)	6 (4.11)	30 (20.55)	110 (75.34)
Sleep quality				
Good/Very good	919 (86.45)	10 (1.09)	100 (10.88)	809 (88.03)
Other (moderate, poor, very poor)	144 (13.55)	12 (8.33)	44 (30.56)	88 (61.11)
ADL score, mean (SD)	1.14 (0.37)	1.12 (0.38)	1.09 (0.28)	1.15 (0.38)
Cognitive functioning score, mean (SD)	4.60 (1.19)	4.55 (1.13)	4.63 (1.20)	4.60 (1.19)

The majority of respondents (84.3%) reported getting lengthy amounts of sleep, followed by those who said they got medium amounts of sleep (13.5%), and then those who said they got short amounts of sleep (2.1%). Respondents who were male, between the ages of 60 and 69, lived in rural areas, were currently married, had completed secondary education, were in the lowest income quintile, had normal weight, and reported having good quality sleep were among those who reported having a lengthy sleep. A similar pattern was observed among those who did not have a history of stroke, diabetes, hypertension, or depression, as well as among individuals who had never used tobacco or alcohol.

The findings of an ordered logistic regression are shown in Table 2, which investigates the relationship between social capital and sleep duration. There was a consistent finding across all models of a negative and statistically significant link between social participation and short sleep duration. The results of the medium and long sleep duration analysis did not show this pattern. After adjusting for all factors in Model 4, there was a positive correlation between sleep quality and long sleep duration among older adults. Our study found no correlation between social capital measures and medium sleep duration. On the other hand, there were significant connections found between the medium amount of time spent sleeping and one’s location of residence, body mass index, hypertension, sleep quality, and ADL. The results for long sleep duration were the same as those for medium sleep: there was no significant connection between these two variables and any of the measures of social capital. Similar to medium sleep duration, there was a statistically significant connection between having a long sleep duration and one’s location of residence, body mass index, hypertension, quality of sleep, and ADL.

**TABLE 2:** Ordered logistic regression analysis of the relationship between social capital and sleep duration (World Health Organization Study on Global AGEing and Adult Health, Ghana, 2014–2015).

	Model 1 Coef. [95% CI]	Model 2 Coef. [95% CI]	Model 3 Coef. [95% CI]	Model 4 Coef. [95% CI]
Short sleep				
Trust	0.006 [−5.26–0.539]	−0.124 [−0.679–0.431]	−0.159 [−0.720–0.402]	−0.193 [−0.766–0.379]
Social participation	−0.629 [−1.149 to −0.108] **	−0.632 [−1.159 to −0.104] **	−0.595 [−1.119 to −0.072]*	−0.412 [−0.981 to −0.155] *
Social Support	−1.436 [−3.115–0.242]	−1.388 [−3.079–0.303]	−1.339 [−3.018–0.338]	−1.473 [−3.182–0.236]
Gender		−0.474 [−1.515–0.567]	−0.250 [−1.392–0.891]	−0.212 [−1.442–1.017]
Age		−0.517 [−1.099–0.063]	−0.491 [−1.074–0.092]	−0.366 [−0.988–0.255]
Residence		−0.519 [−1.435–0.396]	−0.573 [−1.527–0.381]	−0.587 [−1.572–0.396]
Marital status		0.332 [−0.040–0.705]	0.308 [−0.06–0.681]	0.264 [−0.117–0.647]
Education		−0.012 [−0.373–0.349]	−0.010 [−0.371–0.351]	0.020 [−0.360–0.401]
Household income		−0.170 [−0.461–0.120]	−0.166 [−0.458–0.125]	−0.102 [−0.408–0.203]
Tobacco intake			0.096 [−1.47–1.664]	0.242 [−1.366–1.852]
Alcohol intake			−0.629 [−1.562–0.304]	−0.587 [−1.569–0.395]
BMI			0.001 [−0.536–0.537]	0.117 [−0.441–0.677]
Stroke				13.306 [−24.891–24.5]
Diabetes				0.661 [−1.539–2.863]
Hypertension				−0.331 [−1.508–0.846]
Depression				13.349 [−36.546–36.24
Sleep quality				2.019 [1.101–2.937] ***
ADL				0.025 [−1.267–1.318]
Cognition				−55.787 [−88.723–87.151]
Medium sleep				
Trust	0.054 [−0.183–0.291]	0.044 [−0.200–0.288]	−0.010 [−0.259–0.238]	−0.090 [−0.350–0.169]
Social participation	0.022 [−0.182–0.226]	−0.002 [−0.211–0.206]	−0.005 [−0.214–0.202]	0.061 [−0.160–0.284]
Social Support	−0.239 [−0.610–0.131]	−0.226 [−0.603–0.149]	−0.191 [−0.568–0.185]	−0.217 [−0.613–0.178]
Gender		0.011 [−0.408–0.430]	−0.045 [−0.508–0.417]	−0.131 [−0.619–0.355]
Age		−0.184 [−0.410–0.042]	−0.163 [−0.392–0.065]	−0.114 [−0.361–0.132]
Residence		−0.552 [−0.921 to −0.183] **	−0.426 [−0.810 to −0.041] *	−0.465 [−0.868 to −0.063] *
Marital status		−0.098 [−0.254–0.062]	−0.100 [−0.262–0.061]	−0.149 [−0.320–0.022]
Education		−0.102 [−0.254–0.049]	−0.108 [−0.260–0.043]	−0.107 [−0.267–0.053]
Household income		0.042 [−0.074–0.158]	0.043 [−0.073–0.160]	0.060 [−0.061–0.182]
Tobacco intake			−0.510 [−1.130–0.108]	−0.366 [−1.015–0.282]
Alcohol intake			−0.080 [−0.471–0.310]	0.037 [−0.371–0.447]
BMI			0.307 [0.083–0.532] **	0.305 [0.066–0.544] **
Stroke				0.033 [−1.366–1.433]
Diabetes				−0.016 [−0.824–0.790]
Hypertension				−0.594 [−1.087 to −0.102] **
Depression				−1.321 [−2.833–0.191]
Sleep quality				1.466 [1.017–1.916] ***
ADL				−0.770 [−1.471 to −0.069] *
Cognition				−0.070 [−0.242–0.101]
Long sleep				
Trust	0.044 [−0.178–0.267]	0.014 [−0.214–0.244]	−0.038 [−0.272–0.194]	−0.125 [−0.372–0.122]
Social participation	−0.072 [−0.266–0.121]	0.334 [−0.296–0.100]	−0.096 [−0.294–0.101]	−0.010 [−0.224–0.202]
Social Support	−0.357 [−0.723–0.008]	−0.341 [−0.711–0.029]	−0.305 [−0.675–0.064]	−0.365 [−0.756–0.025]
Gender		−0.062 [−0.460–0.336]	−0.080 [−0.519–0.358]	−0.170 [−0.638–0.297]
Age		−0.238 [−0.453 to −0.023] *	−0.217 [−0.434 to −0.001] *	−0.166 [−0.403–0.070]
Residence		−0.580 [−0.929 to −0.231] ***	−0.474 [−0.838 to −0.110]**	−0.527 [−0.913 to −0.140] **
Marital status		−0.033 [−0.183–0.116]	−0.038 [−0.189–0.113]	−0.087 [−0.249–0.073]
Education		−0.090 [−0.233–0.052]	−0.095 [−0.238–0.048]	−0.095 [−0.249–0.057]
Household income		0.011 [−0.098–0.121]	0.013 [−0.096–0.123]	0.038 [−0.078–0.155]
Tobacco intake			−0.451 [−1.042–0.139]	−0.302 [−0.932–0.327]
Alcohol intake			−0.181 [−0.549–0.186]	−0.061 [−0.451–0.328]
BMI			0.278 [0.066–0.490] **	0.293 [0.063–0.522] **
Stroke				0.245 [−1.162–1.653]
Diabetes				0.055 [−0.740–0.851]
Hypertension				−0.618 [−1.094 to −0.141] **
Depression				−1.109 [−2.634–0.415]
Sleep quality				1.726 [1.296–2.156] ***
ADL				−0.687 [−1.326 to −0.048] *
Cognition				−0.069 [−0.233–0.094]

The findings of the model are shown as Beta coefficients, 95% confidence intervals (CIs), and the *p*-value.

*p < 0.05; **p < 0.01; ***p < 0.001.

Model 1: Trust + Social Participation + Social Support.

Model 2: Trust + Social Participation + Social Support + Gender + Age + Residence + Marital status + Education + Household income.

Model 3: Trust + Social Participation + Social Support + Gender + Age + Residence + Marital status + Education + Household income + Tobacco intake + Alcohol intake + BMI.

Model 4: Trust + Social Participation + Social Support + Gender + Age + Residence + Marital status + Education + Household income + Tobacco intake + Alcohol intake + BMI + Stroke + Diabetes + Hypertension + Depression + Sleep quality + ADL + Cognitive function.

## Discussion

In this study, we found that only social participation, as one of the measures of social capital, has a significant negative impact on short sleep duration among older adults. What this suggests is that worse social participation was cross-sectionally associated with an increased risk of short sleep, regardless of other relevant sociodemographic and behavioral covariates. In addition, older adults who do not participate in social activities showed the strongest association with the risk of short sleep. On the other hand, no significant associations between measures of social capital and medium and long sleep durations were observed.

Multiple factors may contribute to the relationship between low social participation and a greater risk of short sleep among older adults; a) loneliness: older adults with minimal social engagement may feel loneliness and depression, which may disrupt their sleep; b) boredom: older adults who do not participate in many social activities may suffer boredom and a lack of stimulation during the day, making it harder for them to fall asleep at night; c) inactivity: older adults with little social engagement are likely to be less physically active, which can also affect the quality of their sleep; d) chronic health issues: inadequate social involvement can increase the prevalence of chronic health disorders in older adults, such as arthritis or heart disease, and these illnesses can also impair sleep quality and length; e) Medication: older adults with minimal social engagement may be more prone to use sleep-inducing medication, which can have adverse side effects and become habit-forming over time.

Our findings significantly contribute to the growing body of research linking social participation with aging. Sleep deprivation is now widely recognized as a behavioral risk factor for healthy aging by epidemiologists [37–39]. Social participation, especially religious interaction, has been shown to reduce mortality and illness among the elderly in several studies [37–39]. Nevertheless, the processes through which social participation reduces illness or death are not fully known. Considering the correlation between quality sleep and longevity in later life, it’s reasonable to assume that social participation has an impact on health outcomes in older adults.

Few studies have examined the prospective relationship between measures of social capital and sleep duration in older adults [21–26], even though the fact that sleep problems are more common in the elderly than in younger adults [40], and that both short and long sleep in the elderly is associated with worse cognitive performance [41, 42], as well as increased likelihood of depression [43], falls [3], and impairment [4]. These findings cannot be directly compared to ours because they assessed different social connections and employed other measures of sleep duration, but they similarly established a significant effect of social capital on sleep outcomes. Similar to our study, in a sample of 1,417 older adults in Singapore, those with a poor social network had an elevated risk of sleeplessness [25]. In addition, two more studies [23] established that an increase in social participation was not related to better actigraphic measures of sleep quality and duration.

The study also observed a significant positive association between sleep quality and short sleep. A significant positive relationship between sleep quality and short sleep duration shows that those who sleep for shorter durations tend to report higher sleep quality. However, this association is likely to be complicated and can be affected by a variety of variables, including age, lifestyle, and health state. Although some people may be able to function effectively on less sleep, the majority of experts recommend 7–9 h of sleep every night for optimal health [44]. Chronic short sleep duration has been linked to adverse health effects, such as cardiovascular disease, obesity, and depression [45]. The optimal amount of sleep varies between individuals and may be impacted by age and health behaviors. People who obtain sufficient sleep report better sleep quality than those who do not.

Long sleep was shown to have a significant negative correlation with age. The cause of the significant negative correlation between age and long sleep duration is unclear, and there are some possible explanations. It is generally seen that older adults sleep less than younger individuals, which may indicate a negative correlation between age and sleep duration [46]. Changes in sleep patterns and demands, medical disorders, and lifestyle variables such as retirement, caregiving obligations, and increased daytime activities can all contribute to alterations in sleep patterns [47]. It is crucial to highlight that correlations do not always imply causality and further study is needed to determine the underlying processes driving this link.

Place of residence, hypertension, and activities of daily living were significantly and negatively correlated with medium and long sleep durations. Individuals with difficulty doing everyday tasks or who suffer from hypertension may choose to sleep for shorter durations [48]. Additionally, BMI had a negative correlation with medium and long sleep durations, as individuals with higher BMI may suffer from sleep apnea or other sleep-related respiratory issues [49]. Sleep apnea can lead to several nighttime awakenings, necessitating additional sleep to feel refreshed during the day.

Understanding how people from different backgrounds approach and experience social capital is essential for understanding the association between social contacts and health outcomes [50–52]. According to the Social-Ecological Model of Sleep Health [12, 53], social-contextual elements that underlie health in general and sleep health, in particular, are the root cause of sleep problems. This work investigates various aspects of social capital and the connection between those aspects and the amount of time spent sleeping by older adults.

A potential policy implication of the relationship between low social participation and a high risk of short sleep duration among older adults in Ghana may be addressed through community-building efforts and programs. This may involve collaborating with local groups and community leaders to provide activities, events, and opportunities for older individuals to engage with one another. Additionally, providing older adults with access to healthcare services, promoting good sleep habits, and educating healthcare practitioners and policymakers on the importance of addressing sleep health may help reduce the risk of short sleep duration and enhance their health and wellbeing.

Utilizing a nationally representative sample of older adults is a strength of this study because it allows for the results to be generalized to the larger population of older adults in the country. This increases the external validity and generalizability of the study’s findings and reduces the potential for selection bias. The use of self-reported sleep quality and sleep duration, which may be impacted by recollection and social desirability biases, is a limitation of our study. Due to their low cost and ease of use, self-reported measures of sleep are often used in large prospective studies. Although objective measures of sleep duration are more reliable, self-reported data are the only way to obtain information on an individual’s assessment of sleep quality. In addition, the translation of the questionnaire from English to other local languages may introduce concerns about reliability.

### Conclusion

In line with the findings of this study, previous research has consistently demonstrated that older adults who engage in fewer social activities and have limited social connections are more prone to experiencing sleep-related difficulties, such as trouble initiating sleep, maintaining sleep, and early morning awakening. Several factors may contribute to this association, including heightened stress and anxiety levels, reduced physical activity, and disruptions in circadian rhythms. Consequently, it can be concluded that promoting social interaction and reducing social isolation among older adults may have a positive impact on their sleep quality. Implementing interventions such as community programs, enhancing healthcare accessibility, fostering robust social support networks, and encouraging healthy sleep habits are potential strategies to address the correlation between older adults’ limited social participation and short sleep duration.
