# Supplementary material for: The Relationship Between Social Capital and Sleep Duration Among Older Adults in Ghana: A Cross-Sectional Study
Source: Int J Public Health. 2023 Jun 29;68:1605876. doi: 10.3389/ijph.2023.1605876 (PMC10338686; doi:10.3389/ijph.2023.1605876)
Supplement: Supplementary file 1 [file DataSheet1.PDF]

Supplementary File

Questions about social capital measures and recoding

SOCIAL PARTICIPATION

Thank you for your comment. To recode the questions on social participation with a binary "yes" or "no" response in STATA, we will explain the recoding process for each question, including how the "yes" or "no" response relates to the original responses.

Q1 - How often in the last 12 months have you attended any public meeting in which there was discussion of local or school affairs?

Original Responses:

1. NEVER
2. ONCE OR TWICE PER YEAR
3. ONCE OR TWICE PER MONTH
4. ONCE OR TWICE PER WEEK
5. DAILY

Recoding: To recode this question into a binary "yes" or "no" response, we considered responses 1 and 2 as "no" (indicating infrequent attendance) and responses 3, 4, and 5 as "yes" (indicating regular attendance).

Q2 - How often in the last 12 months have you met personally with someone you consider to be a community leader?

Original Responses:

1. NEVER
2. ONCE OR TWICE PER YEAR
3. ONCE OR TWICE PER MONTH
4. ONCE OR TWICE PER WEEK

5. DAILY

Recoding: To recode this question, we considered responses 1 and 2 as "no" (indicating infrequent meetings with community leaders) and responses 3, 4, and 5 as "yes" (indicating regular meetings).

Q3 - How often in the last 12 months have you attended any group, club, society, union or organizational meeting?

Original Responses:

1. NEVER
2. ONCE OR TWICE PER YEAR
3. ONCE OR TWICE PER MONTH
4. ONCE OR TWICE PER WEEK
5. DAILY

Recoding: For this question, responses 1 and 2 were recoded as "no" (indicating infrequent attendance), while responses 3, 4, and 5 were recoded as "yes" (indicating regular attendance).

Q4 - How often in the last 12 months have you worked with other people in your neighborhood to fix or improve something?

Original Responses:

1. NEVER
2. ONCE OR TWICE PER YEAR
3. ONCE OR TWICE PER MONTH
4. ONCE OR TWICE PER WEEK
5. DAILY

Recoding: Responses 1 and 2 were recoded as "no" (indicating infrequent collaboration), while responses 3, 4, and 5 were recoded as "yes" (indicating regular collaboration).

Q5 - How often in the last 12 months have you had friends over to your home?

Original Responses:

1. NEVER
2. ONCE OR TWICE PER YEAR
3. ONCE OR TWICE PER MONTH
4. ONCE OR TWICE PER WEEK
5. DAILY

Recoding: For this question, responses 1 and 2 were recoded as "no" (indicating infrequent social gatherings), while responses 3, 4, and 5 were recoded as "yes" (indicating regular social gatherings).

Q6 - How often in the last 12 months have you been in the home of someone who lives in a different neighborhood than you do or had them in your home?

Original Responses:

1. NEVER
2. ONCE OR TWICE PER YEAR
3. ONCE OR TWICE PER MONTH
4. ONCE OR TWICE PER WEEK
5. DAILY

Recoding: Responses 1 and 2 were recoded as "no" (indicating infrequent interactions with people from different neighborhoods), while responses 3, 4, and 5 were recoded as "yes" (indicating regular interactions).

Q7 - How often in the last 12 months have you socialized with coworkers outside of work?

Original Responses:

1. NEVER
2. ONCE OR TWICE PER YEAR
3. ONCE OR TWICE PER MONTH
4. ONCE OR TWICE PER WEEK
5. DAILY

Recoding: For this question, responses 1 and 2 were recoded as "no" (indicating infrequent socialization with coworkers), while responses 3, 4, and 5 were recoded as "yes" (indicating regular socialization).

Q8 - How often in the last 12 months have you attended religious services (not including weddings and funerals)?

Original Responses:

1. NEVER
2. ONCE OR TWICE PER YEAR
3. ONCE OR TWICE PER MONTH
4. ONCE OR TWICE PER WEEK
5. DAILY

Recoding: Responses 1 and 2 were recoded as "no" (indicating infrequent religious service attendance), while responses 3, 4, and 5 were recoded as "yes" (indicating regular attendance).

Q9 - How often in the last 12 months have you gotten out of the house/your dwelling to attend social meetings, activities, programs or events or to visit friends or relatives?

Original Responses:

1. NEVER
2. ONCE OR TWICE PER YEAR
3. ONCE OR TWICE PER MONTH
4. ONCE OR TWICE PER WEEK
5. DAILY

Recoding: Responses 1 and 2 were recoded as "no" (indicating infrequent outings), while responses 3, 4, and 5 were recoded as "yes" (indicating regular outings).

SOCIAL SUPPORT

Q10 - First, how often do you feel that you lack companionship?

Original Responses:

1. Never
2. Rarely
3. Sometimes
4. Often

Recoding: To recode the responses into a binary "yes" or "no" variable, we considered responses 1 and 2 as "no" (indicating sufficient companionship) and responses 3 and 4 as "yes" (indicating a lack of companionship).

Q11 - How often do you feel left out?

Original Responses:

1. Never
2. Rarely
3. Sometimes
4. Often

Recoding: For this question, we recoded responses 1 and 2 as "no" (indicating not feeling left out) and responses 3 and 4 as "yes" (indicating feeling left out).

Q12 - How often do you feel isolated from others?

Original Responses:

1. Never
2. Rarely
3. Sometimes
4. Often

Recoding: Responses 1 and 2 were recoded as "no" (indicating not feeling isolated) and responses 3 and 4 as "yes" (indicating feeling isolated).

TRUST

Q13 - Generally speaking, would you say that most people can be trusted or that you can't be too careful in dealing with people?

Original Responses:

1. CAN BE TRUSTED
2. CAN'T BE TOO CAREFUL

Recoding: For this question, we recoded response 1 as "yes" (indicating that most people can be trusted) and response 2 as "no" (indicating that one can't be too careful in dealing with people).

Q14 - Do you have someone you can trust and confide in?

Original Responses:

1. Yes
2. No

Recoding: We retained the original responses for this question without recoding since it already represents a binary "yes" or "no" variable. Response 1 was interpreted as "yes" (having someone to trust and confide in), and response 2 was interpreted as "no" (lacking someone to trust and confide in).

Q15 - First, think about people in your neighborhood. Generally speaking, would you say that you can trust them…?

Original Responses:

1. To a very great extent

2. To a great extent
3. Neither great nor small extent
4. To a small extent
5. To a very small extent

Recoding: To create a binary "yes" or "no" variable, we considered responses 1, 2, and 3 as "yes" (indicating trust to some extent) and responses 4 and 5 as "no" (indicating lack of trust).

Q16 - Now, think about people whom you work with. Generally speaking, would you say that you can trust them …?

Original Responses:

1. To a very great extent
2. To a great extent
3. Neither great nor small extent
4. To a small extent
5. To a very small extent

Recoding: Similar to Q15, we recoded responses 1, 2, and 3 as "yes" (indicating trust to some extent) and responses 4 and 5 as "no" (indicating lack of trust).

Q17 - And how about strangers? Generally speaking, would you say that you can trust them?

Original Responses:

1. To a very great extent
2. To a great extent
3. Neither great nor small extent
4. To a small extent
5. To a very small extent

Recoding: Similarly, we recoded responses 1, 2, and 3 as "yes" (indicating trust to some extent) and responses 4 and 5 as "no" (indicating lack of trust).
